# Predictors of urinary tract infection in acute stroke patients: A cohort study

**DOI:** 10.1097/MD.0000000000020952

**Published:** 2020-07-02

**Authors:** Ya-ming Li, Jian-hua Xu, Yan-xin Zhao

**Affiliations:** a Department of Neurology, Jiading District Central Hospital affiliated to Shanghai University of Medicine & Health Sciences; b Department of Neurology, Tenth People's Hospital affiliated to Tongji University, Shanghai, China.

**Keywords:** hemoglobin, infection, interleukin-6, prediction, stroke, urinary tract infection

## Abstract

Patients with stroke have a high risk of infection which may be predicted by age, procalcitonin, interleukin-6, C-reactive protein, National Institute of Health stroke scale (NHSS) score, diabetes, etc. These prediction methods can reduce unfavourable outcome by preventing the occurrence of infection.

We aim to identify early predictors for urinary tract infection in patients after stroke.

In 186 collected acute stroke patients, we divided them into urinary tract infection group, other infection type groups, and non-infected group. Data were recorded at admission. Independent risk factors and infection prediction model were determined using Logistic regression analyses. Likelihood ratio test was used to detect the prediction effect of the model. Receiver operating characteristic curve and the corresponding area under the curve were used to measure the predictive accuracy of indicators for urinary tract infection.

Of the 186 subjects, there were 35 cases of urinary tract infection. Elevated interleukin-6, higher NIHSS, and decreased hemoglobin may be used to predict urinary tract infection. And the predictive model for urinary tract infection (including sex, NIHSS, interleukin-6, and hemoglobin) have the best predictive effect.

This study is the first to discover that decreased hemoglobin at admission may predict urinary tract infection. The prediction model shows the best accuracy.

## 1 Introduction

Stroke has become the disease with the first disability and the second mortality rate in the world.^[1]^ Ischemic stroke accounts for 87% of all stroke patients.^[2]^ Stroke can cause immunosuppression and the transfer and ectopic of specific intestinal flora, which makes stroke patients more susceptible to be infected.^[3,4]^ The concept of post-stroke infection (PSI) was defined by Vargis^[5]^ in 2006. After that, Emsley and Hopkrns^[6]^ supplemented this concept which mainly referred to the infection that occurred 48 hours after the onset of stroke. In addition, the infection was not in its occurrence or incubation when a stroke occurred. PSI mainly includes stroke-associated pneumonia (SAP) and urinary tract infection (UTI). The probability of infection after stroke is about 25% to 65%.^[7]^ And, SAP has a greater effect on prognosis than UTI.^[8]^ However, studies of SAP have been relatively mature. By summarizing a large number of previous studies, the predictors of SAP include multiple vertebrobasilar stroke, National Institutes of Health Stroke Scale score, mechanical ventilation, nasogastric tube use, and dysphagia.^[9]^ However, there are relatively few studies on urinary tract infections. The incidence of urinary tract infection is about 19%.^[10]^ In addition, the occurrence of infection can further aggravate the physical damage caused by stroke, and this process will form a vicious circle with stroke.^[11–16]^ The circle will lead to a worse clinical prognosis.^[7,17–20]^

Therefore, the prevention and treatment of post-stroke infection are particularly critical. In previous studies, preventive antibiotic therapy did not improve functional outcome in relatively unselected patients with stroke.^[21–24]^ On the contrary, actively searching for signs of infection and prophylactic use of antibiotics were beneficial for patients at high risk of infection.^[25–28]^ In 1997, Fassbender found that interleukin-6 (IL-6) might be feasible in the prediction of post-stroke infection, which was the earliest study on the prediction of post-stroke infection.^[29]^ In later studies, risk factors for post-stroke infection had been found to include higher age, procalcitonin (PCT), interleukin-6, C-reactive protein (CRP), higher NIHSS (National Institute of Health stroke scale) score at admission, diabetes, etc.^[6,30–32]^

In this study, we attempted to identify early predictors and construct a prediction model which is simple and practical for urinary tract infection in patients with acute ischaemic stroke.

## 2 Material and methods

### 2.1 Patient population

A total of 186 patients with acute ischemic stroke admitted to the stroke unit of the department of neurology of Shanghai Tenth Peopleʼs Hospital from June 2014 to December 2016 were continuously collected. Patients were enrolled in the study if they

(1) had an acute-onset focal neurological deficit combined with neuroimaging evidence of cerebral infarction by cranial computed tomography or magnetic resonance imaging,
(2) were hospitalized within 48 hours after onset of stroke symptoms,
(3) were not in the infection incubation or occurrence phase at the onset of stroke,
(4) had no signs of infection within 48 hours after the onset of stroke, and
(5) gave informed consent.

Patients were excluded from the study if they

(1) had an intracranial hemorrhage, hypoglycemia, or other causes of a new focal deficit,
(2) had severe liver, kidney, and heart dysfunctions,
(3) were being treated with antibiotics, immunosuppressors, or corticosteroids in the previous 3 months and significant disability before the index stroke,
(4) had a history of surgery or trauma within a month,
(5) had immunodeficiency or malignant tumors,
(6) had diseases of the blood system or serious lung diseases, and
(7) had been placed into the catheter.

### 2.2 Clinical management and data

The stroke patients were admitted to a dedicated stroke unit. The neurological course was assessed using the NIHSS score^[33]^ and the Oxfordshire Community Stroke Project classification^[34]^ by neurologists at admission. On the first day of hospitalization, clinical and demographic data of the patients, including age, sex, and vascular risk factors (arterial hypertension, diabetes mellitus, atrial fibrillation, hyperlipidemia, and smoking status) were recorded. Every patient's fasting venous blood was sampled on the second day of admission. The venous blood was used to examine laboratory indicators such as interleukin-6, procalcitonin, etc. In addition, all patients completed the examination of stroke and post-stroke infection after admission.

### 2.3 Outcome measures

UTI was defined as urinary tract symptoms and positive midstream urine culture results (growth of bacteria > 105 colony forming units/mL and no more than 2 microbial species).^[35]^ Other infections were diagnosed according to the diagnostic criteria for the corresponding diseases. After the infection was diagnosed, we divided the infected patients into urinary tract infection group, other infection type groups, and non-infected group.

### 2.4 Statistical analysis

Data were sorted out and statistically analyzed using SPSS (Statistical Product and Service Solutions) software package version 22.0 (IBM Corp, Armonk, NY, USA). Continuous variables that conformed to a normal distribution were expressed as means ± standard deviations. If the continuous variables did not fit the normal distribution, they were represented by *M* (*Q*25-*Q*75). The 2 groups of continuous variables subject to normal distribution were compared by T test (Student *t* test). Wilcoxon signed-rank test was used for 2 groups of measurement data that did not conform to normal distribution. Differences in categorical data between groups were examined using Pearson's Chi-squared test. Independent risk factors were determined using multivariate Logistic regression analyses. Infection prediction model was established by binary Logistic stepwise regression analyses. After that, Likelihood ratio test was used to detect the prediction effect of the model. Receiver Operating Characteristic (ROC) curves and the corresponding area under the curve were used to measure the predictive accuracy of indicators and the model for urinary tract infection. *P* (probability) < .05 was considered statistically significant in all these tests.

## 3 Results

### 3.1 Baseline characteristics

A total of 186 patients were included in the study, including 127 males, accounting for 68.28%, and 59 females, accounting for 31.72%. No patients fell off from observational trials. Among the subjects, the oldest was 95 years old, the youngest was 30 years old, and the average age was 66.88 ± 11.87 years old. Of the 186 subjects in the study, 64 were infected. These data were calculated during the hospital stay. The incidence of post-stroke infection was 34.41%. Among the infections, there were 23 cases of pulmonary infection, accounting for 35.94%; 32 cases of urinary tract infections, accounting for 50.00%; 6 cases of other infections (5 cases of acute upper respiratory tract infection, 1 case of acute periodontitis), accounting for 9.38%; 3 cases of pulmonary infection combined with urinary tract infection, accounting for 4.69%.

### 3.2 Urinary tract infection group

#### 3.2.1 Indicators of patients with and without urinary tract infection group

A total of 35 cases of urinary tract infection occurred in 186 patients. The incidence rate was 18.82%. Comparison of indicators related to patients with and without urinary tract infection occurred in Table 1. Indicators that accounted for a higher proportion in the infected group than those in the uninfected group included female, no smoking history, higher admission NIHSS score. In the laboratory test results, the levels of interleukin-6 and procalcitonin in patients with urinary tract infection were higher than those in patients without infection, and the levels of hemoglobin in patients with urinary tract infection were lower than patients without infection.

**Table 1:**
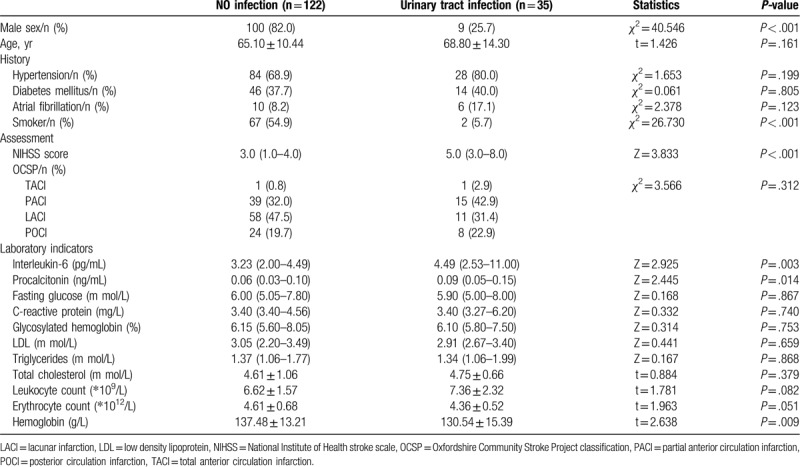
Univariate associations of factors influencing urinary tract infection.

#### 3.2.2 Independent influencing factors of UTI

Multivariate Logistic regression analysis showed that sex, smoking, NIHSS score, interleukin-6, and hemoglobin were independent influencing factors of urinary tract infection. And, if the NIHSS score and interleukin-6 levels were higher, the risk of infection was greater. However, if the levels of hemoglobin were higher, the risk of infection was lower. Smoking history was a protective factor of urinary tract infection. Conversely, female was a risk factor for urinary tract infection. (Table 2)

**Table 2:**
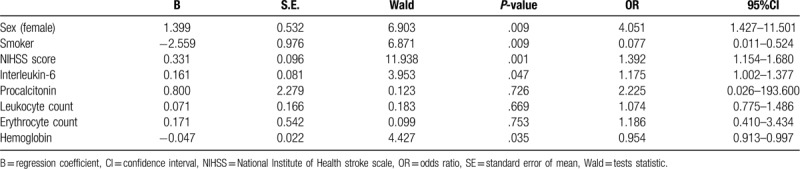
Multivariate logistic regression analysis of factors influencing UTI.

#### 3.2.3 Establishment and analysis of UTI prediction model

The index with *P*-value less than .10 in the comparison between the infected patients and the non-infected patients was included in the logistic regression model, and the forward method was used to perform stepwise regression. The model standard was *P* < .05, and the exclusion criterion was *P* > .10. The variables that eventually entered the model were sex, NIHSS score, interleukin-6, and hemoglobin. (Table 3)

**Table 3:**
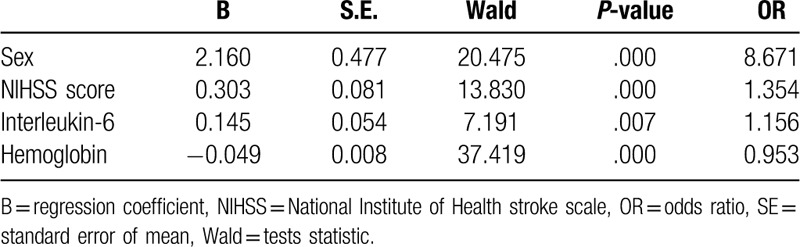
Logistic regression model results.

Then we assigned the variables that entered the regression model. The assignment table was shown in Table 4.

**Table 4:**
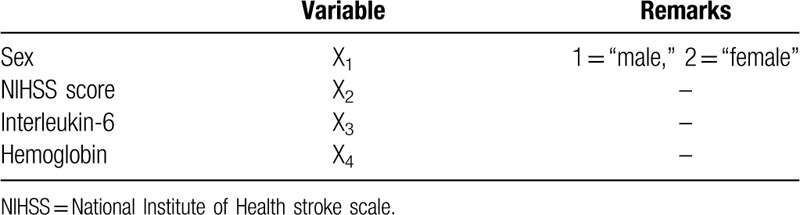
Variable assignments.

The variables that finally entered the model were sex (X_1_), NIHSS score (X_2_), interleukin-6 (X_3_), and hemoglobin (X_4_).

The regression model was: Logit (*P*) =2.160X_1_+0.303X_2_+0.145X_3_–0.049X_4_.

Next, the model was tested for likelihood ratio, and the test result showed that the regression model was statistically significant (*χ*^2^ = 116.894, *P* < .001). The prediction of infection in the urinary tract infection prediction model showed that of the 35 patients with urinary tract infection, 17 were correctly predicted; 122 were patients with no infection, and 115 were correctly predicted. The accuracy of the regression model prediction was 85.4%.

#### 3.2.4 Predictive diagnostic value of different indicators for UTI

**Figure 1:**
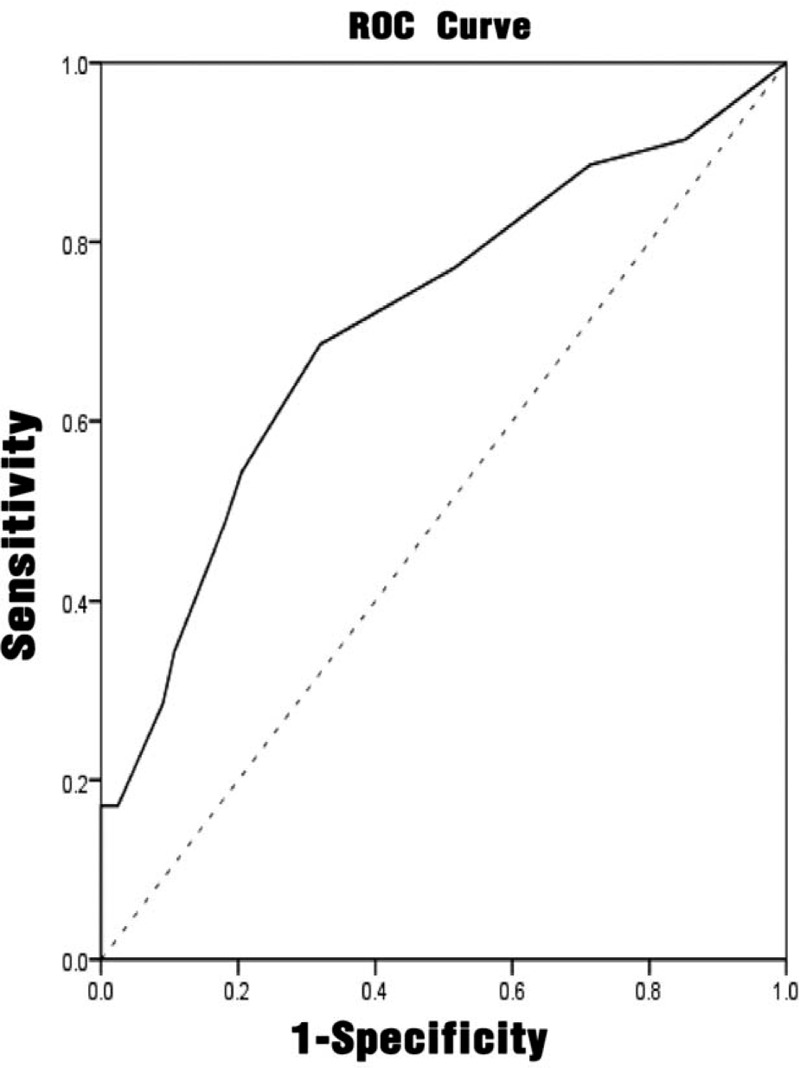
ROC curve of the NIHSS score for predicting the accuracy of urinary tract infection. NIHSS = national institute of health stroke scale, ROC = receiver-operating characteristic.

**Figure 2:**
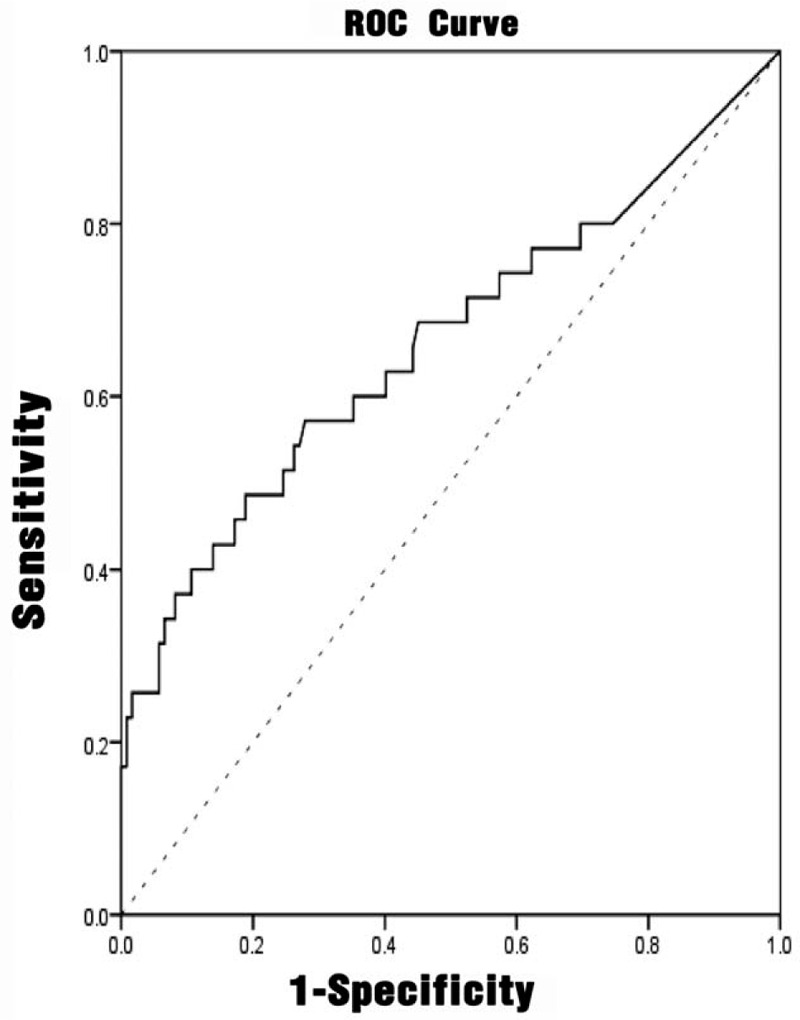
ROC curve of interleukin-6 for predicting the accuracy of urinary tract infection. ROC = receiver-operating characteristic.

**Figure 3:**
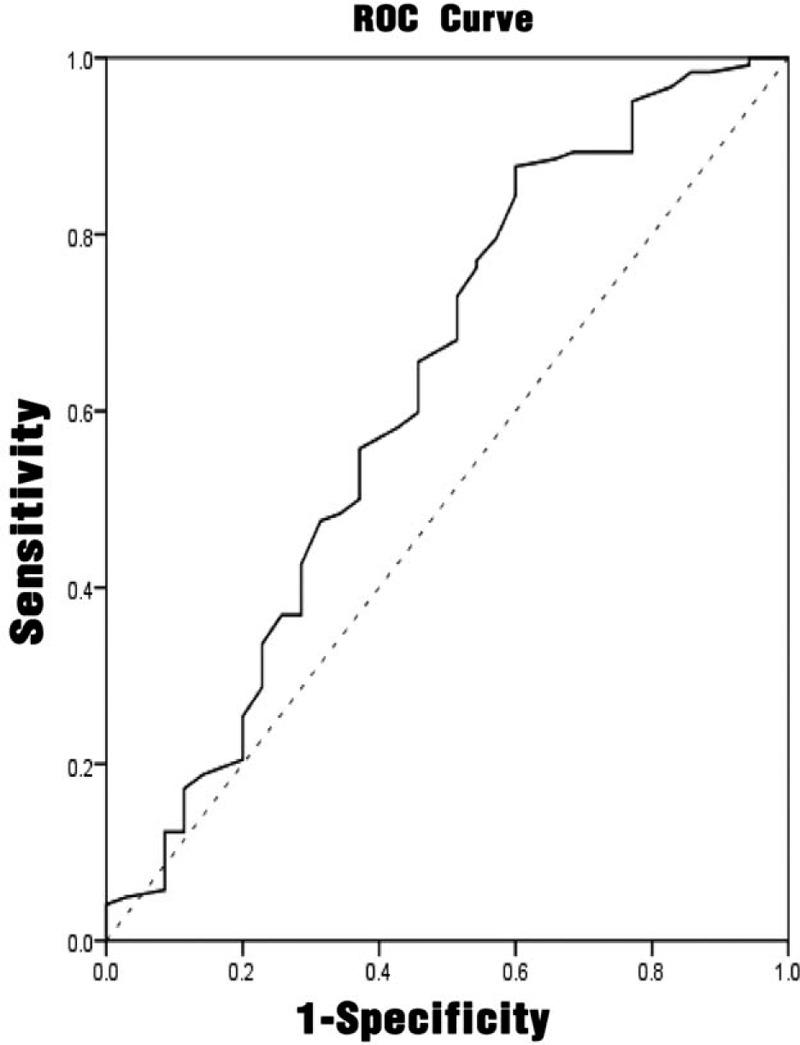
ROC curve of hemoglobin for predicting the accuracy of urinary tract infection. ROC = receiver-operating characteristic.

**Figure 4:**
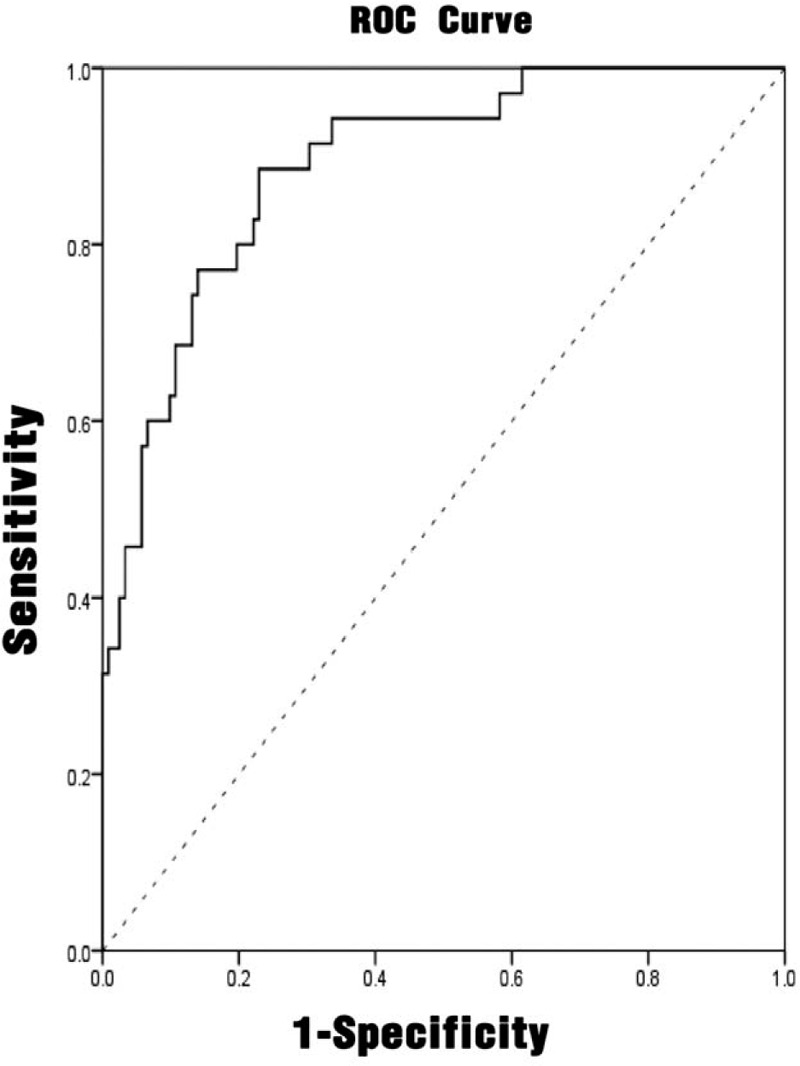
ROC curve of the predictive model for predicting the accuracy of urinary tract infection. ROC = receiver-operating characteristic.

Multivariate analysis had shown that NIHSS score, interleukin-6, and hemoglobin were independent influencing factors affecting urinary tract infection. The ROC analyses were performed on the NIHSS score, interleukin-6, procalcitonin, and urinary tract infection prediction model, as shown in Figures 1–4. The results showed that different predictors and the predictive model all could predict the occurrence of urinary tract infection (*P* < .05). The area under the ROC curve of NIHSS score, interleukin-6, and hemoglobin for urinary tract infection was 0.711 (95% CI: 0.607 to 0.815), 0.661 (95% CI: 0.546 to 0.777), and 0.625 (95% CI: 0.510 to 0.740), respectively. Furthermore, NIHSS score at a cutoff value of 3.5 exhibited the best balance between sensitivity and specificity for detection of urinary tract infection, followed by interleukin-6 (cutoff value, 4.910 pg/mL) and hemoglobin (cutoff value: 123.50 g/L). The area under the ROC curve of the predictive model of UTI was 0.890 (95% CI: 0.832 to 0.948). When the probability of regression model *P*≥.2014 was predicted to occur, the sensitivity at this time was 88.57%, and the specificity was 77.05%. (Table 5)

**Table 5:**
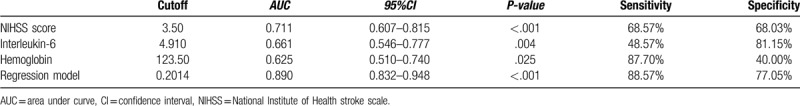
Predictive effects of different indicators on urinary tract infection.

## 4 Disscussion

### 4.1 Hemoglobin

This study is the first to discover that the decreased hemoglobin levels at admission may predict the occurrence of UTI. Hemoglobin is a protein responsible for carrying oxygen in an organism.^[36]^ Decreased hemoglobin content in the blood will result in a relative decrease in oxygen supply to the local tissues and organs, which may result in a decrease in the metabolism of tissues and organs, thereby facilitating secondary infection. The previous study has found that bacterial infections were associated with hemoglobin levels.^[37]^ Moreover, Eneroth's research has found that patients with anemia were prone to infection, suggesting that reduced hemoglobin might be a predictor of infection.^[38]^ In addition, Kotze's study has found that low-grade inflammation was inversely related to hemoglobin content, which meant that the lower the hemoglobin content, the higher the likelihood of an inflammatory response.^[39]^ The mechanism of this phenomenon may be that hemoglobin can naturally decompose in red blood cells, and then some larger fragments are generated and secreted into the blood. These fragments are further broken down into smaller fragments which form a “hemoglobin peptide library” in different tissues. The “hemoglobin peptide library” can produce different biological effects which include “antimicrobial hemoglobin-derived peptides.” Then, the “antimicrobial hemoglobin-derived peptides” can produce antibacterial effects, thereby reducing inflammation caused by microbial infections.^[40]^

### 4.2 Interleukin-6, NIHSS score, and sex

Our study found that elevated levels of interleukin-6 and higher admission NIHSS score might be used as independent risk factors to predict UTI.

Interleukin-6 is a cytokine produced by monocytes, macrophages, lymphocytes, and so on, and belongs to the class of interleukins. It is an important mediator of the acute phase of inflammation. And, it will rise in the acute phase of inflammation.^[41]^ Bacterial infections can induce normal cells to produce interleukin-6. Subsequently, the interleukin-6 will stimulate the proliferation and differentiation of cells involved in the immune response, thereby enhancing the function of these cells. Finally, through this process, interleukin-6 plays an anti-infective role.^[42]^The results of our study found that interleukin-6 might be used for the prediction of UTI after a stroke is about the same as previous studies.^[16,17,29]^

And, C-reactive protein and procalcitonin are also inflammatory factors. Do they also have a certain relationship with interleukin-6? Then, we statistically analyzed the data of all patients and reached the following conclusions. C-reactive protein was positively correlated with interleukin-6 (*r* = 0.343, *P* < .001). There was no correlation between C-reactive protein and procalcitonin. Interleukin-6 was positively correlated with procalcitonin (*r* = .217, *P* = .006). Therefore, we searched in detail for the reasons for the above phenomena, and listed them below.

C-reactive protein is a marker of inflammation and its levels increase during bacterial infection.^[43]^ Some studies have found that there is a correlation between elevated interleukin-6 levels and elevated C-reactive protein levels when inflammation occurs in the body, which may be because interleukin-6 can induce the C-reactive protein gene.^[44]^ Procalcitonin is a 116 amino acid prohormone of calcitonin. When a bacterial infection occurs, the CALC-1 gene is up-regulated, which results in a large amount of procalcitonin produced by monocytes and macrophages throughout the body. Then, procalcitonin can be measured within 2 to3 hours and reaches a maximum at 6 hours. Therefore, it is very sensitive to bacterial infections. However, procalcitonin can also be elevated in various non-infectious diseases (e.g., surgery, burns, fracture, myocardial infarction, pancreatitis, renal failure). Moreover, the diagnostic value for local infections (e.g., endocarditis, empyema) is also poor.^[45]^ Instead, Interleukin-6 is more sensitive to local infections.^[46]^ And, it responds more quickly to inflammatory reactions than procalcitonin and C-reactive protein.^[46,47]^ After an infection in a stroke patient, the body experiences similar reactions as described above. And our data statistics show that procalcitonin and interleukin-6 are positively correlated, which seems to be consistent with the above results. In addition, for patients who are immunosuppressed, their C-reactive protein will not increase much even if a serious bacterial infection occurs.^[45]^ And, procalcitonin responds faster to inflammation than C-reactive protein, due to cytokine like behavior. C-reactive protein can be detected at 4 to 6 hours and reaches its maximum at 36 to 50 hours.^[48]^ Because the blood of the patients we tested was taken the morning after admission. This phenomenon may explain that the C-reactive protein did not differ significantly between infected and non-infected groups in this study (*P* = .740). And it may be the reason that C-reactive protein and procalcitonin are not related after statistical analysis.

In addition, previous studies have found a correlation between interleukin-6 and stroke prognosis.^[49]^ And, the ratio of neutrophils to lymphocytes (NLR) is also related to stroke prognosis.^[50–53]^ Moreover, NLR can reflect the body's immune level when a stress reaction occurs.^[54,55]^ When a stroke occurs, the body will be in an immunosuppressed state, making it more susceptible to infection. These findings led us to speculate whether NLR is related to UTI. Unfortunately, through statistical data, it was found that NLR did not show statistical differences (Z = 1.291, *P* = .197) between infected and non-infected groups.

The NIHSS score can assess the severity of a patient's stroke to some extent and can roughly predict the size of the stroke area.^[56]^ Previous research found that brain injury after an ischaemic stroke could lead to immunosuppression^[3]^ which had been related to the increased risk of infection after stroke.^[57]^ Moreover, the severity of stroke is a risk factor for post-stroke immunosuppression.^[58]^ And, a large number of studies have previously demonstrated that higher NIHSS score at admission could be used for post-stroke infection predictions.^[14,32,59–61]^ The results were about repeated in our trial.

Our study has found that female was an independent risk factor for urinary tract infections. The reason is caused by the particularity of the structure of the female genitourinary system. Women's urethra is shorter than men, which is more conducive to bacterial invasion. Moreover, the female urethra is close to the vagina and anus which contain a lot of bacteria. And, vaginal secretions are also a good medium for bacteria to multiply. These conditions can be used to explain that women are more likely to get a urinary tract infection. At the same time, previous research has reached the same conclusion.^[62]^

Further, previous studies have found that post-stroke infections can worsen stroke prognosis.^[19]^ There are many factors that affect the prognosis of stroke. The factors include: NIHSS score,^[63]^ age, a measure of stroke severity, prestrike function, comorbidities and stroke subtype, acute glucose, history of atrial fibrillation, congestive heart failure, cancer, kidney disease, preadmission dependency, and so on.^[64]^ And, a review summarized the blood biomarkers that could predict the prognosis of stroke. They were sirtuin1,^[65]^ serum retinoic,^[66]^ interleukin-6, tumor necrosis factor alpha, C-reactive protein, cholesterol, high density lipoprotein cholesterol (HDL-C) and low density lipoprotein (LDL), glutamate, glutamic oxaloacetic transaminase, glutamic-pyruvic transaminase, brain-derived neurotrophic factor, and fibrinogen.^[67]^

Among these factors, perfusion and hemodynamic disturbances, inflammatory response, metabolic homeostasis, and drug effects play important roles.

When a stroke occurs in the body, different perfusion and hemodynamic disturbances can cause different stroke types and severity. In the anterior circulation, the middle cerebral artery produces a subcortical infarction due to branch occlusion. Although a larger area of infarction may be associated with cortical dysfunction, its clinical symptoms are similar to lacunar syndrome associated with small perforator artery diseases. Thromboembolism in situ of the anterior circulation artery leads to a larger infarction, which leads to cortical symptoms. In the posterior circulation, post-cerebral atherosclerosis produces pure midbrain or thalamus infarction through branch occlusion. Artery to artery embolisms lead to cortical infarction – cerebellar or temporo-occipital lobe infarction.^[68]^ And, Cerebral infarction caused by insufficient blood perfusion is less severe than vascular occlusion.^[69]^ Moreover, insufficient blood perfusion is associated with vascular stenosis and changes in blood pressure. Previous research found that systolic blood pressure variability in stroke patients might be related to poor prognosis.^[70]^ Therefore, combined with the above discussion, we believe that the different stroke types and severity caused by perfusion and hemodynamic disturbances may have a considerable impact on stroke outcome.

After a stroke occurs, the body will produce an inflammatory response through a series of reactions. Specifically, ischemia and hypoxia of brain tissue due to interruption of blood flow can cause the blood-brain barrier to lose parts of its leukocyte-mediated barrier properties. This allows endothelial cell to induce selectins and integrins, and secrete proinflammatory mediators, such as tumor necrosis factor alpha, interleukin-6, monocyte chemoattractant protein-1, etc. Brain ischemia and hypoxia can also cause swelling and detachment of brain endothelial cells, resulting in increased protein extravasation and interstitial edema. This process can cause the adhesion of leukocytes and the increase of reactive oxygen species, resulting in the peroxidation of cell membranes, leading to an increase in vascular permeability and angioedema, and ultimately aggravating brain tissue damage. Early after reperfusion of the central nervous system, white blood cells begin to accumulate in the capillaries of the brain and may cause microthrombosis, resulting in insufficient perfusion downstream. These damage methods not only directly affect neuronal function, but can also lead to the activation of local and systemic inflammatory responses. Moreover, oxidative stress and mitochondrial dysfunction can cause excitatory toxic reactions of neurons. This reaction can not only aggravate the damage of the nervous system, but also further drive local and systemic immune responses. In addition, the activation of local inflammatory response after ischemic central nervous system injury also depends on innate immune response. Specifically, central nervous system damage first triggers the activation of microglia, macrophages, neutrophils, dendritic cells, and other monocytes. These cells then migrate from the bone marrow to the central nervous system. This process is also involved in the activation of complement and platelet activation. Among them, the activation of complement and the activation of platelets can lead to persistent inflammatory imbalances and brain tissue damage, and drive local and systemic inflammatory responses. This inflammatory response also exacerbates the ischemic brain environment, which further changes the behavior of immune cells.^[71]^ In the above process, a large number of inflammatory factors are produced and involved. And, some inflammatory factors have been found to be related to the prognosis of stroke. They were Ficolin-1,^[72]^ interleukin-6, tumor necrosis factor alpha, C-reactive protein, cholesterol, HDL-C, and LDL, etc. Moreover, previous studies have also found that the prognosis of stroke was related to the inflammatory response.^[73,74]^

The above inflammatory factors include substances produced by lipid metabolism. Previous studies have found that lipid metabolism was associated with stroke prognosis. Especially, low total cholesterol and low HDL-C level was related to poor outcomes in patients with stroke.^[75,76]^ Moreover, circulating high density lipoprotein (HDL) can effectively protect LDL from the oxidative damage of free radicals, thereby inhibiting the production of pro-inflammatory oxidized lipids. In addition, HDL-C can protect cell function through anti-oxidation and reduce atherosclerosis.^[77]^ Previous studies found that blood glucose had an impact on the prognosis of stroke.^[78,79]^ Blood sugar can rise significantly early in the onset of stroke. This may be because stroke can activate the hypothalamic-pituitary-adrenal axis and produce a wide range of stress responses, which subsequently leads to increased serum glucocorticoid levels, sympathetic autonomic nervous system activation and increased catecholamine release. This process can increase glucose production and inhibit insulin-mediated glycogen production. Acute hyperglycemia can also increase the production of cerebral lactic acid, reduce the recovery of cerebral tissue in the ischemic penumbra, and lead to a larger final infarct size. At the same time, elevated blood sugar can also increase reperfusion injury by increasing oxidative stress, stimulating systemic inflammation, and increasing barrier permeability. In addition, high blood sugar can increase the aggregation and adhesion of platelets. Therefore, diabetes is related to poor stroke prognosis.^[79]^ There is also a study which found that metabolic syndrome complicated with diabetes was associated with poor prognosis of stroke.^[80]^ In the early stages of stroke, dysfunction of ion transporters and disrupted ionic homeostasis occur in brain tissue. In the process of ischemia-induced cell damage, The Na^+^/K^+^-ATPase, transient receptor potential (TRP) channels, Na^+^/Ca^2+^ exchanger, and ionotropic glutamate receptor play an important role. They can induce cytotoxic edema, excitotoxicity, necrosis, apoptosis, and autophagic cell death.^[81]^ In addition, serum magnesium is associated with stroke prognosis.^[82]^ Magnesium ions has been recognized as a cofactor for > 300 metabolic reactions in the body. It can participate in the synthesis of various substances and the production and storage of cellular energy. It can also stabilizes the mitochondrial membrane. And, magnesium plays a key role in vasomotor contraction, blood pressure regulation, glucose and insulin metabolism. Therefore, magnesium ion has a great role in preventing stroke recurrence and improving stroke prognosis in stroke patients.^[83]^ The above-mentioned lipid metabolism, sugar metabolism, energy metabolism, and ion metabolism are all related to the prognosis of stroke. Therefore, maintaining metabolic homeostasis is very important for improving the prognosis of stroke.

The use of anti-platelet aggregation drugs^[84]^ and statins^[85,86]^ for patients with ischemic stroke can improve the prognosis of patients. For patients with hemorrhagic stroke, dehydration to reduce intracranial pressure and proper blood pressure control can improve the prognosis of patients.^[87]^ And, for all stroke patients, reasonable control of blood pressure is of great significance to improve the prognosis of patients.^[88]^ Because high blood sugar can worsen the prognosis of stroke, the use of hypoglycemic drugs can reduce the morbidity and mortality of patients.^[89]^ In addition, a review summarized new drugs that might have therapeutic effects, and the therapeutic mechanism of these drugs was explained in parentheses. These drugs were uric acid (reduced excessive intracellular calcium and excitotoxicity), resveratro (prevented oxidative stress and attenuated neuronal death), coumestrol (neuroprotective and prevented long-term neuronal death), fumarate (improved functional outcome and decreased edema volume after stroke), necrostatin-1 (inhibited necroptosis, suppressed apoptosis and autophagy, reduced brain edema and blood–brain barrier disruption, and improved neurological outcome), etc.^[90]^ Besides, post-stroke infection can worsen the prognosis of stroke patients. Thus, the use of drugs that can prevent the occurrence of infection after stroke is very meaningful for improving the prognosis of stroke patients. This requires the use of prophylactic antibiotics. Therefore, the infection prediction model obtained by this research may improve the prognosis of stroke patients by predicting urinary tract infections after stroke.

### 4.3 Smoking

Interestingly, our study found that smoking history was a protective factor for urinary tract infection after ischemic stroke. The phenomenon of protective effects on smoking was first discovered in the field of heart disease. In coronary myocardial infarction, patients with a history of smoking had a lower incidence, mortality, and myocardial reinfarction rate than those without a history of smoking. The study also found that no smoking history was an independent risk factor for myocardial infarction recurrence.^[91]^ Moreover, after an acute myocardial infarction, smokers exhibited a better clinical outcome than patients who have never smoked. In addition, coronary angiography showed that the area of coronary artery lesions in smokers was smaller.^[92]^ However, the above study only evaluated the history of smoking at admission and did not conduct subsequent assessments. It is speculated that the sudden cessation of smoking after admission may be used to explain the phenomenon of lower recurrent myocardial infarction and a better prognosis in patients with a history of smoking.^[93,94]^

Later, some studies also found that smokers showed a better prognosis in acute myocardial infarction.^[95–97]^ Novo summarized previous studies and speculated that possible causes of good prognosis in hospitalized patients with acute myocardial infarction included:

(1) younger and less associated disease;
(2) higher pre-hospital mortality;
(3) smoking was more likely to cause myocardial infarction caused by thrombosis, which made patients obtain a better thrombolytic effect;
(4) smoking could cause a protective effect similar to ischemic preconditioning.

Moreover, he described the phenomenon of ischemic preconditioning in his research. It referred to a transient ischemic stimulus which gave cardiomyocytes better tolerance to subsequent ischemic events. The protective effect of ischemic preconditioning depended on functional channels of gap junction intercellular communication, which were specialized intercellular contacts that allowed electrical impulse propagation among cardiomyocytes. The main structure of the gap junction was composed of connexin 43, which played a major role in ischemic preconditioning. In addition, smoking could induce gap junction remodeling of cardiomyocytes, thereby increasing the function of gap junctions. And, the enhanced function could better protect cells. This phenomenon might explain the better prognosis in patients with myocardial infarction who had a history of smoking.^[98]^

Previous research found that gap junctions existed between cells of various tissues, except for blood cells and skeletal muscle cells. Moreover, they were widely present between urinary tract cells, bronchial epithelial cells, alveolar epithelial cells, alveolar macrophages, smooth muscle cells, and pulmonary artery endothelial cells. The gap junction could help the cilia to clear the mucus, facilitate the secretion of alveolar surfactant, and promote the synchronous contraction of pulmonary vascular smooth muscle cells. It could also fight the lung inflammation and may even have a therapeutic effect on lung inflammation.^[99]^ Besides, connexin 43 could increase pulmonary vascular permeability, which was more conducive to anti-inflammatory cells to fight infection.^[100]^ So we speculate that gap junctions in urinary tract cells may also exert anti-inflammatory effects in the same way.

Since previous studies have found that smoking had a protective effect on the damage caused by acute myocardial infarction, we have reason to speculate that smoking may exert anti-inflammatory effects by inducing remodeling of gap junctions. Moreover, isoflurane pretreatment can reduce the release of proinflammatory factors in the lungs and the mortality caused by sepsis.^[101]^ Pretreatment with sevoflurane and isoflurane can reduce the body's inflammatory response and pneumonia damage caused by sepsis.^[102]^ Cigarette smoke contains alkane components. It is speculated that smoking may have a similar pretreatment effect. Smoking thus has a similar protective effect on ischemic stroke infection. Cigarette smoke can also increase the function of macrophages to fight the infection caused by Leishmania donovani.^[103]^ More importantly, stroke patients will suddenly quit smoking after admission, which may also be the reason why the probability of urinary tract infection in stroke patients with smoking history found in this study is low. Besides, it was found that among all stroke patients included in our study, the age of smokers was significantly lower than that of non-smokers (*P* < .05). The immunity and general condition of younger people may be better than those of the elderly, and past diseases may be less, resulting in a lower risk of infection. This finding may also be the reason why smoking is a protective factor for infection after ischemic stroke.

## 5 Conclusion

In summary, our study is the first to discover that the decreased hemoglobin levels at admission may predict the occurrence of UTI. Besides, elevated levels of interleukin-6 and higher NIHSS score at admission may also be used as independent risk factors to predict UTI. Moreover, the prediction models of UTI have the best predictive effect. The predictive model for UTI included risk factors for sex (female), higher NIHSS score, elevated interleukin-6 levels, and reduced hemoglobin levels. And, the prediction model established in our study may provide a reference for the prediction of stroke prognosis.

This study has some limitations. First, the number of cases included in this study is not large enough, and the conclusions obtained may not fully reflect the overall situation. There may be bias. Second, this study did not include all characteristics reported in previous studies as possible risk factors for infection, such as interleukin-10, interleukin-1 receptor antagonist, etc. Third, this study did not further observe the guiding role of these predictive models for antibiotic prophylaxis. Subsequent studies should include larger samples and further observation of the clinical effect of the predictive model for prophylactic antibiotic use. Furthermore, it would be very meaningful to establish a similar prediction model to predict the prognosis of stroke patients. Therefore, this study may contribute to the establishment of a stroke prognosis prediction model in the future.

## Author contributions

Ya-ming Li wrote the first draft of the manuscript and gathered data. Jian-hua Xu statistically processed the data. Yan-xin Zhao conceived the research and modified the manuscript. Jian-hua Xu was involved in data analyses. All authors reviewed and edited the manuscript and approved the final version of the article.
